# Repurposing memantine as an oral therapy for visceral leishmaniasis: identification of direct leishmanicidal activity and immune system modulation in preclinical studies

**DOI:** 10.3389/fphar.2026.1761504

**Published:** 2026-03-30

**Authors:** Gabriella Gonçalves-Ozório, Larissa G. Capilla, Yago S. S. Emiliano, Luiza F. O. Gervazoni, Paula M. De Luca, Elmo E. Almeida-Amaral

**Affiliations:** 1 Laboratório de Bioquímica de Tripanosomatídeos, Instituto Oswaldo Cruz, Fundação Oswaldo Cruz, Rio de Janeiro, Brazil; 2 Laboratório de Imunoparasitologia, Instituto Oswaldo Cruz, Fundação Oswaldo Cruz, Rio de Janeiro, Brazil

**Keywords:** immunomodulation, memantine, oral treatment, repurposing, visceral leishmaniasis

## Abstract

**Introduction:**

Leishmaniasis, a neglected tropical disease caused by *Leishmania spp*., affects millions of individuals worldwide. Visceral leishmaniasis which represents the most severe disease type, is fatal if untreated. Current treatments are associated with various challenges, making drug repurposing a practical alternative. This study evaluated the NMDA receptor antagonist memantine for the treatment of *Leishmania infantum*.

**Methods:**

To assess the effect of memantine against amastigote forms, peritoneal macrophages from BALB/c mice were infected with *Leishmania infantum* promastigotes, and the proportion of infected macrophages was assessed via light microscopy and expressed as an infection index. In a murine model of visceral leishmaniasis, the efficacy of memantine was evaluated by using two different treatment schemes (short-term and long-term), and the parasite load in the liver and spleen was quantified by using a limiting dilution assay. Meglumine antimoniate, which is the reference drug used in clinical settings for leishmaniasis treatment, was selected as the positive control. The immunological profile of uninfected and infected BALB/c mice (either treated or not treated with memantine or the reference drug) was evaluated in splenocyte cultures (with or without stimulation with the *Leishmania infantum* antigen).

**Results:**

*In vitro*, memantine demonstrated significant activity against intracellular amastigotes (IC_50_ = 5.49 ± 0.11 μM; SI = 603.64). *In vivo*, the oral administration of memantine (3 and 6 mg/kg/day) in BALB/c mice reduced the parasite burden in the liver and spleen by >99%, thereby outperforming lower doses and conventional meglumine antimoniate treatment. Parasite clearance was consistent with nitrosative stress-independent immunomodulation and was associated with a shift towards a Th1-dominant immune response (involving increased IFN-γ/IL-10 levels), in addition to Th2 and Th17 activation (including IL-2, IL-4, and IL-17), thus promoting parasite control and granuloma formation. Memantine was observed to be safe and well-tolerated; additionally, it demonstrated immunomodulatory effects by rebalancing the immune response to favor parasite clearance.

**Conclusion:**

Memantine exhibits dual effects, including direct antiparasitic activity and host-directed immunomodulation. These findings support its potential use as a repurposed candidate for treating visceral leishmaniasis and reinforce the value of drug repositioning as a strategy to accelerate the development of safe and effective therapies.

## 1 Introduction

Leishmaniasis, a neglected tropical disease, represents a significant global health challenge, affecting more than 1 billion people across 99 countries. It is closely associated with poverty and limited resources in tropical and subtropical regions. Cutaneous leishmaniasis (CL) accounts for approximately 95% of cases in the Americas, the Mediterranean Basin, the Middle East, and Central Asia, with an estimated 600,000 to 1 million new cases reported annually. Visceral leishmaniasis (VL), which is the most severe form of the illness, is endemic in 80 countries, with India, Sudan, Brazil, and Kenya accounting for 68% of cases. VL, if untreated, can be fatal because of its systemic progression, primarily affecting vital organs such as the spleen and liver and causing bone marrow damage (Costa et al., 2023; World Health Organization, 2023).

The success of *Leishmania* infection depends on the parasite’s ability to alter the host’s immune system. The initial interaction at the site of a sand fly bite triggers an inflammatory cascade. The parasite uses proinflammatory molecules from the sand fly saliva to evade early immune defenses. Macrophages, the primary cellular defense, are modulated by *Leishmania*, which induces and inhibits cytokine expression, alters phagocytic activity, and interferes with antigen recognition and presentation (Rossi and Fasel, 2018; Atri et al., 2018; Costa-da-Silva et al., 2022).

Despite its impact, leishmaniasis remains neglected, with limited investments in research and development. Current treatments are problematic, costly, and associated with severe side effects such as nephrotoxicity, hepatotoxicity, and cardiotoxicity, making them unsuitable for some patients, especially those with comorbidities such as HIV/AIDS (World Health Organization, 2023). The emergence of drug-resistant *Leishmania* strains further complicates treatment, highlighting the urgent need for safer, more affordable, and effective alternatives (Aït-Oudhia et al., 2011). Drug repurposing, which is a strategy used for identifying new therapeutic uses for approved drugs, represents a promising solution due to reduced development risks and established safety profiles (Langedijk et al., 2015; Andrade-Neto et al., 2018). Repurposed drugs account for nearly 30% of new drug and vaccine approvals by the FDA and are cost effective, especially for neglected tropical diseases (Krisnamurthy et al., 2022). Examples of treatments for leishmaniasis include pentamidine, amphotericin B, miltefosine, and paromomycin (Berenstein et al., 2016).

Memantine (1-amino-3,5-dimethyladamantane), which is a low-affinity, voltage dependent, noncompetitive antagonist of N-methyl-D-aspartate receptors (NMDARs), is a candidate for repurposing (Ettcheto et al., 2018). It was initially synthesized in the 1960s as a potential antidiabetic agent, and its effects on the central nervous system (CNS) were recognized in 1972, leading to its use to treat neurological disorders. By 1989, it was identified as an NMDA receptor antagonist for glutamate (Gerzon et al., 1963; Wesemann et al., 1983).

Memantine is widely used to treat Alzheimer’s disease because of its neuroprotective effects on glutamate-induced excitotoxicity. In addition to its effects on neurodegeneration, it exhibits immunomodulatory properties. In murine models of neuroinflammation, sepsis-associated encephalopathy, and autoimmune disorders, memantine reduced microglial activation and decreased the expression of proinflammatory cytokines (TNF-α, IL-1β, and IL-6), exerting both central and systemic anti-inflammatory effects (Willard et al., 2000; Hao et al., 2021). Memantine can selectively modulate Th1/Th17 pathways and influence the expression of cytokines (IFN-γ, IL-12, and IL-10) depending on the pathological context (Van Dyk et al., 2020). The induction of IL-10 production by memantine has been reported to limit immunopathology without compromising pathogen clearance (Rosi et al., 2006).

Memantine has also been shown to affect Trypanosomatidae, inhibit *Trypanosoma cruzi* proliferation, interfere with metacyclogenesis, disrupt parasite energy metabolism, and induce apoptosis-like mechanisms. It also affects the intracellular cycle during the amastigote stage (Damasceno et al., 2014). Analogs of amantadine and memantine have been shown to have trypanocidal effects on *Trypanosoma brucei* (Duque et al., 2010). However, its leishmanicidal potential has not been investigated.

Given the global relevance of leishmaniasis and current treatment limitations, in this study, the effectiveness of memantine against *Leishmania infantum* intracellular amastigotes and in a murine VL model were evaluated via a drug repositioning approach, and the involvement of the host immune response in shaping its therapeutic effect was investigated.

## 2 Materials and methods

### 2.1 Compounds and reagents

Memantine hydrochloride was obtained from Tocris Bioscience (Bristol, UK). Schneider’s *Drosophila* medium, RPMI-1640 medium, penicillin, streptomycin, ketamine, xylazine, and concanavalin A (ConA) were obtained from Merck/Sigma-Aldrich (Darmstadt, Germany). The Cytometric Bead Array kit (CBA) was obtained from BD Bioscience (San Jose, CA, USA). Fetal calf serum was obtained from Cultilab (Campinas, SP, Brazil). Memantine was diluted in RPMI-1640 medium (amastigote assay) or phosphate-buffered saline (PBS) (*in vivo* experiments).

### 2.2 Ethics statement

This study was conducted in strict accordance with the guidelines established by the Brazilian National Council for the Control of Animal Experimentation (CONCEA) for the care and use of laboratory animals. All the animals were bred and maintained at the Oswaldo Cruz Foundation (FIOCRUZ) according to CONCEA regulations. The experimental protocol was approved by the Ethics Committee on Animal Use of the Instituto Oswaldo Cruz (CEUA-IOC, License Number: L-010/2024).

### 2.3 Parasites and animals

For this study, promastigotes of *Leishmania infantum* (strain MHOM/MA/67/ITMAP263) were maintained at 26 °C in Schneider’s *Drosophila* medium (pH 6.9). The culture medium was supplemented with 20% (v/v) fetal calf serum, 100 U/mL penicillin, and 100 μg/mL streptomycin; moreover, the parasites were subcultured every 3 days. The animal model involved female BALB/c mice aged 8–10 weeks, which were supplied by the Instituto de Ciências e Tecnologia em Biomodelos (ICTB/FIOCRUZ).

### 2.4 Cytotoxicity assay

To evaluate the cytotoxicity of memantine, peritoneal macrophages were harvested and allowed to adhere for 1 h. Subsequently, the cells were exposed to a range of memantine concentrations (ranging from 3.9 μM to 8 mM) for 72 h at 37 °C in a 5% CO_2_ incubator (without the presence of parasites). Cell viability was quantified by using the Alamar blue assay. Fluorescence was measured on a spectrofluorometer with excitation set at 560 nm and emission at 590 nm. The 50% cytotoxic concentration (CC_50_) was calculated from the resulting data using non-linear regression in GraphPad Prism 6 (GraphPad Software, La Jolla, CA, USA). Each experimental condition was performed in triplicate.

### 2.5 Leishmania–macrophage intracellular assay

The anti-leishmanial activity of memantine was assessed by using an intracellular assay. Peritoneal macrophages from BALB/c mice were resuspended in RPMI-1640 medium containing 10% fetal calf serum (RPMI/FCS). These cells were seeded onto Lab-Tek eight-chamber slides at a concentration of 2 × 10^6^ cells/mL (0.4 mL per well) and incubated for 1 h at 37 °C in a 5% CO_2_ atmosphere to allow for cell adhesion.

Macrophages were then infected with stationary-phase *L. infantum* promastigotes at a multiplicity of infection (MOI) of 5:1 (parasite-to-cell ratio). The coculture was incubated for 5 h. Following this infection period, non-adherent cells and free promastigotes were removed via washing with RPMI/FCS. Fresh medium supplemented with 2% heat-inactivated horse serum was then added, and the cultures were incubated for another 18 h (Inacio et al., 2019).

The infected macrophages were subsequently treated with various concentrations of memantine (ranging from 4.4 μM to 280 μM) for 72 h. Control cells received only the vehicle treatment (RPMI-1640 medium). After treatment, the slides were stained by using Instant Prov (Newprov, Curitiba/Brazil). The infection index was calculated by examining the slides under a light microscope, counting at least 200 macrophages, and applying the following formula: percentage of infected macrophages × number of amastigotes per macrophage. The 50% inhibitory concentration (IC_50_) was determined by using non-linear regression in GraphPad Prism 6. All of the assays were conducted in triplicate.

### 2.6 *In vivo* infection in a murine model of visceral leishmaniasis and quantification of the parasite load

To assess the *in vivo* efficacy of memantine, an experimental model of visceral leishmaniasis was established. Female BALB/c mice were maintained under specific pathogen-free conditions and organized into seven groups (n = 5 mice per group), including five infected groups and two non-infected controls. Infection was induced via an intraperitoneal injection of 1 × 10^7^ stationary-phase *L. infantum* promastigotes in a 100 µL volume (Inacio et al., 2019).

Therapeutic intervention was initiated 7 days after infection. Two treatment durations (short-term and long-term durations) were investigated, with meglumine antimoniate being administered to the positive control group. Upon completion of the treatment protocols, the mice were euthanized, and the spleens and livers were aseptically removed and weighed.

Each organ was then entirely macerated and homogenized in Schneider’s medium (with 20% FBS) to quantify the parasite burden using a limiting dilution assay (LDA), wherein the number of viable parasites was determined by using the highest dilution demonstrating promastigote growth after 7 days of incubation at 26 °C (Inacio et al., 2019). The design of this *in vivo* protocol was adapted from the methodology proposed by Katsuno et al. for drug discovery in neglected tropical diseases (Katsuno et al., 2015).

### 2.7 Therapeutic schemes (short- and long-term treatments)

Infected female BALB/c mice were divided into seven groups, including a noninfected control group and an infected control group that both received vehicle treatment (PBS) orally via gavage twice daily; a noninfected group that was treated with memantine at 6 mg/kg/day (administered in two doses of 3 mg/kg each); three infected groups that were treated with memantine at 0.75, 1.5, or 3 mg/kg twice daily (corresponding to total daily doses of 1.5, 3, and 6 mg/kg/day, respectively); and a positive control group that was treated with meglumine antimoniate (100 mg/kg/day intramuscularly once per day), as previously described (Inacio et al., 2019). All treatments were initiated at 7 days post-infection and continued for five consecutive days.

For the short-term evaluation, the mice were euthanized on day 14 (2 days after treatment completion), and liver and spleen samples were collected to quantify the parasite load using the limiting dilution assay (LDA), as previously described.

For the long-term evaluation, the mice were euthanized on day 30 (18 days after treatment completion), and liver and spleen samples were collected for LDA and immunological analyses.

### 2.8 Dose conversion used for memantine treatment

The doses employed in the experiments have been previously applied in clinical practice for patients with Alzheimer’s disease (van Marum, 2009) and major depressive disorder (Ferguson and Shingleton, 2007) (Table 1); moreover, they were adapted for the murine model according to the dose conversion equation described by Reagan-Shaw et al. (Reagan-Shaw et al., 2008). This equation establishes the relationship between weight and surface area (human or animal), resulting in the Km factor, as follows:

$$Animal\;dose\;\left(mg/kg\right)=\frac{Human\;Equivalent\;Dose\;\left(HED\right)\left(mg/kg\right)}{\frac{Animal\;Km}{Human\;Km}}$$

**TABLE 1:** Equivalent dosages of memantine.

Clinical dose	Equivalent dose	Clinical dose reference
10 mg/day	1.5 mg/kg/day	van Marum, 2009 (Alzheimer’s disease)
20 mg/day	3 mg/kg/day	van Marum, 2009 (Alzheimer’s disease)
40 mg/day	6 mg/kg/day	Ferguson and Shingleton, 2007 (Major depressive disorder)

The doses employed in the experiments have been previously used in clinical practice for patients with Alzheimer’s disease and were adapted for the murine model according to the dose conversion equation described by Reagan-Shaw et al. (2008).

### 2.9 Toxicological analysis

For toxicological evaluation, blood samples were collected immediately prior to euthanasia. Mice were first deeply anesthetized via an intraperitoneal injection of a ketamine (200 mg/kg) and xylazine (16 mg/kg) solution. Approximately 1 mL of blood was obtained via cardiac puncture.

For hematological analysis, a portion of the blood was transferred to microtubes containing EDTA. The remaining blood was centrifuged to separate the serum, which was used for assessing toxicological markers. Samples of whole blood and serum were collected from all of the experimental groups (including infected and non-infected groups, as well as treated and untreated groups). All of the analyses were performed by using the Clinical Analysis of Laboratory Animals Platform (RPT12C) at the Technological Platforms Network–FIOCRUZ.

### 2.10 Cytokine assay

Spleens from mice subjected to the long-term treatment scheme were gently homogenized through a 70 μm nylon cell strainer (BD Falcon #352350) into 50-mL tubes with a 6-cc syringe plunger. The strainer was rinsed with RPMI/FCS. The resulting cell suspension was adjusted to a final volume of 30 mL with RPMI-1640/FCS and centrifuged at 1,500 rpm for 10 min. The pellet was gently resuspended and incubated with 3 mL of ACK lysis buffer per spleen for 4 min at room temperature to lyse the red blood cells. The volume was subsequently brought to 50 mL with RPMI-1640/FCS, followed by two washing steps. The cells were then resuspended in 5 mL of RPMI-1640/FCS and passed through a nylon strainer to remove residual debris. Viable cells were counted and adjusted to a final concentration of 3 × 10^6^ cells/mL.

For stimulation assays, splenocytes were cultured in 96-well plates at a density of 3 × 10^5^ cells/well and incubated *in vitro* with 20 μg/mL ConA, 50 μg/mL lysate of stationary-phase *L. infantum* promastigotes or left unstimulated as controls. The cells were cultured for 72 h at 37 °C in a humidified atmosphere containing 5% CO_2_. Following the incubation, the culture supernatants were harvested, and the levels of cytokines, including interferon-gamma (IFN-γ), tumor necrosis factor (TNF), and the interleukins IL-2, IL-4, IL-6, IL-10, and IL-17, were determined. The cytokine levels were quantified using a BD Cytometric Bead Array (CBA) Mouse Th1/Th2/Th17 Cytokine Kit (BD Biosciences, San Jose, CA, USA) according to the manufacturer’s instructions.

### 2.11 NOS activity in spleen cell cultures

Nitric oxide (NO) levels in culture supernatants were indirectly measured using Green’s reaction. Briefly, 100 μL of culture supernatants from infected and noninfected spleen cell cultures, either stimulated or unstimulated, as described above, were mixed with an equal volume of Griess reagent. The reagent consisted of 0.1% N-(1-naphthyl)ethylenediamine dihydrochloride, 1% sulfanilamide, and 5% phosphoric acid. After the samples were incubated for 10 min at room temperature, the absorbance was measured at 540 nm with a microplate reader. Nitrite concentrations, as an indirect indicator of NO production, were calculated using a standard curve prepared with serial dilutions of sodium nitrite in distilled water (Ferreira-Paes et al., 2020).

### 2.12 Statistical analysis

The data were analyzed using Student’s t test or analysis of variance (ANOVA), followed by the Mann‒Whitney *post hoc* test with GraphPad Prism 7 (GraphPad Software, La Jolla, CA, USA). The results were considered significant when p ≤ 0.05. The data are presented as the means ± standard errors.

## 3 Results

### 3.1 Memantine reduces the intracellular amastigote load without affecting macrophage viability

**FIGURE 1:**
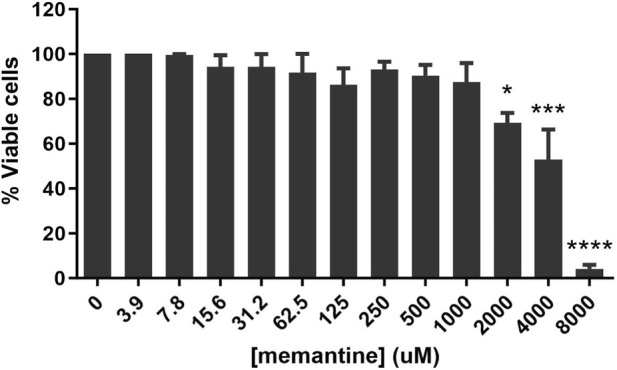
Cytotoxic effect of memantine on murine peritoneal macrophages. Murine peritoneal macrophages were plated and incubated with increasing concentrations of memantine (ranging from 3.9 μM to 8 mM) for 72 h in the absence of infection. Cell viability was assessed using resazurin. The values are presented as the means ± standard errors of three independent experiments (n = 3) performed in triplicate. * indicates a significant difference compared with the control (p ≤ 0.05); ** indicates a significant difference compared with the control (p ≤ 0.01); *** indicates a significant difference compared with the control (p ≤ 0.001); **** indicates a significant difference compared with the control (p ≤ 0.0001).

At concentrations up to 1 mM, memantine was not cytotoxic to noninfected macrophages. A 30.65% reduction in viability was observed only at concentrations higher than 2 mM, with a CC_50_ value of 3.31 ± 0.10 mM after 72 h (Figure 1).

**FIGURE 2:**
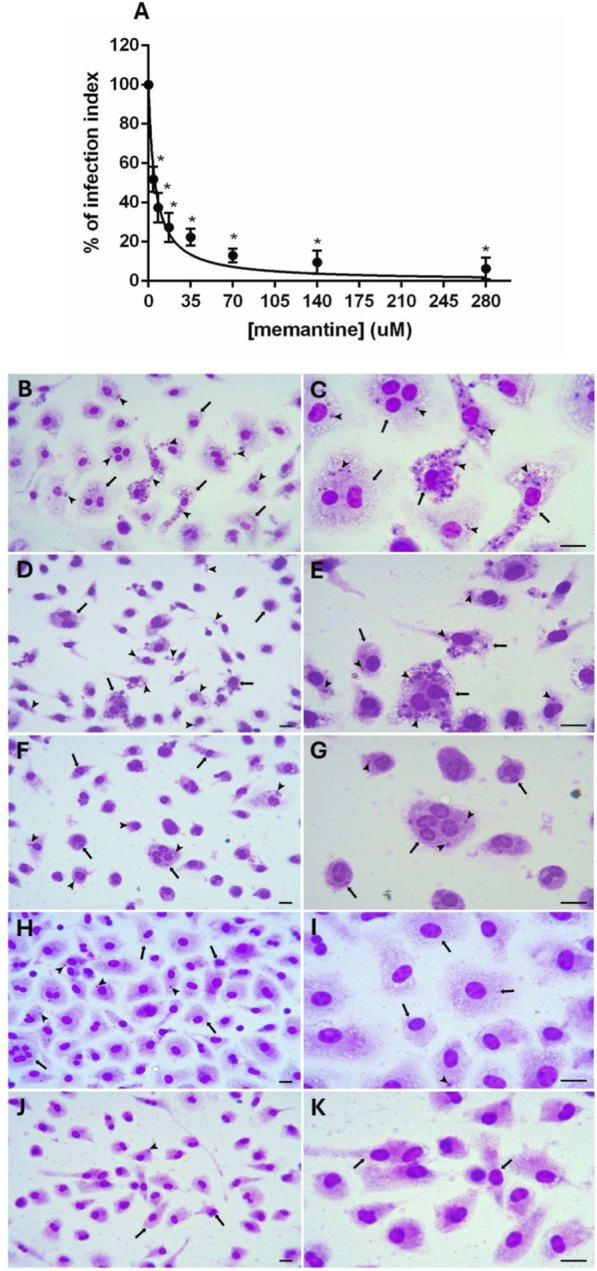
Effect of memantine on *Leishmania infantum-*infected macrophages. **(A)** The infection index was calculated by counting at least 200 macrophages on each duplicated coverslip using light microscopy. All presented values represent the means ± standard errors from three independent experiments (n = 3) performed in duplicate. A significant difference compared with the control is indicated by *p < 0.0001. **(B–K)** Representative images of murine peritoneal macrophages infected with *Leishmania infantum* and incubated with memantine. Images were captured at ×200 (left panel) and ×500 (right panel) magnification. Arrows indicate *Leishmania infantum*-infected macrophages; intracellular amastigotes are indicated by arrowheads. The scale bars correspond to 10 μm. **(B,C)** Control; **(D,E)** 4 μM; **(F,G)** 17 μM; **(H,I)** 70 μM; **(J,K)** 280 μM of memantine.

The *in vitro* effectiveness of memantine against *Leishmania infantum* intracellular amastigotes was evaluated in peritoneal macrophages from BALB/c mice. Infected macrophages were treated with memantine (4.4–280 μM) for 72 h. Memantine significantly reduced the infection index in a concentration-dependent manner, with an IC_50_ of 5.49 ± 0.11 μM and a maximum inhibition of 93.7% at 280 μM (Figure 2A; Supplementary Figure S1). Representative images (Figure 2, panels B–K) further illustrate this effect. The selectivity index (SI), which is defined as the CC_50_/IC_50_ ratio, was 603.64, thus indicating that memantine was not cytotoxic to macrophages at concentrations deemed to be effective against intracellular amastigotes.

### 3.2 Memantine treatment significantly reduces the parasite burden in the liver and spleen of a murine model of visceral leishmaniasis

The *in vivo* activity of memantine was assessed in a murine model of VL by using short-term and long-term therapeutic strategies. In the short-term treatment scheme, *L. infantum*-infected or noninfected BALB/c mice were treated for 5 consecutive days (beginning at 7 days after infection) and euthanized 2 days after treatment (day 14). Under the long-term scheme, euthanasia was delayed until day 30 (18 days after treatment) (Emiliano and Almeida-Amaral, 2023).

**FIGURE 3:**
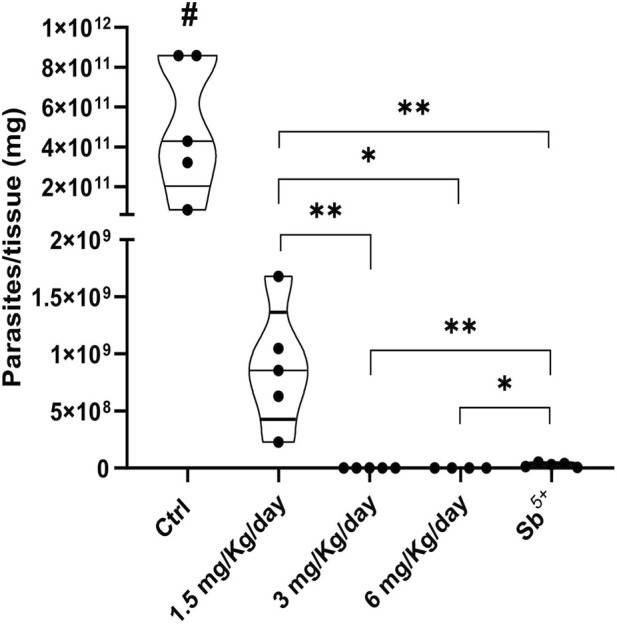
Short-term therapeutic effectiveness of memantine in a murine model of visceral leishmaniasis. BALB/c mice were infected with *Leishmania infantum* promastigotes via intraperitoneal injection. At 7 days post-infection, the mice were treated orally with 1.5, 3 or 6 mg/kg/day of memantine (treated group) or vehicle (control group) or intramuscularly injected with 100 mg/kg/day of meglumine antimoniate (positive control group). After 5 days of treatment (at the end of treatment), the mice were euthanized, and the livers were collected to quantify parasite load via a limiting dilution assay (LDA). A significant difference between control and treated groups is indicated by #p ≤ 0.0001; significant differences between the treated groups are indicated by *p ≤ 0.05 and **p ≤ 0.005. The values are presented as the means ± standard errors of 1 independent experiment with 5 animals per group. Student’s t test with the Mann‒Whitney *post hoc* test were used for the analysis. Ctrl, control; Sb^5+^, meglumine antimoniate.

Short-term oral memantine treatment significantly reduced the liver parasite burden in all of the treated groups, thus resulting in up to 99% inhibition compared with that in the vehicle control group (Figure 3). A trend towards greater efficacy was observed with increasing doses, with the intermediate (3 mg/kg/day) and highest (6 mg/kg/day) doses being more effective than the lowest dose (1.5 mg/kg/day). Compared with meglumine antimoniate, treatment with memantine at both 3 and 6 mg/kg/day resulted in significantly lower parasite burdens. Serological toxicity parameters (total albumin, creatine kinase, urea, alkaline phosphatase, ALT, AST, cholesterol, iron, calcium, sodium, potassium, and total protein levels; Supplementary Table S1) and hematological parameters (Supplementary Table S2) were not significantly altered, indicating the absence of toxicity.

**FIGURE 4:**
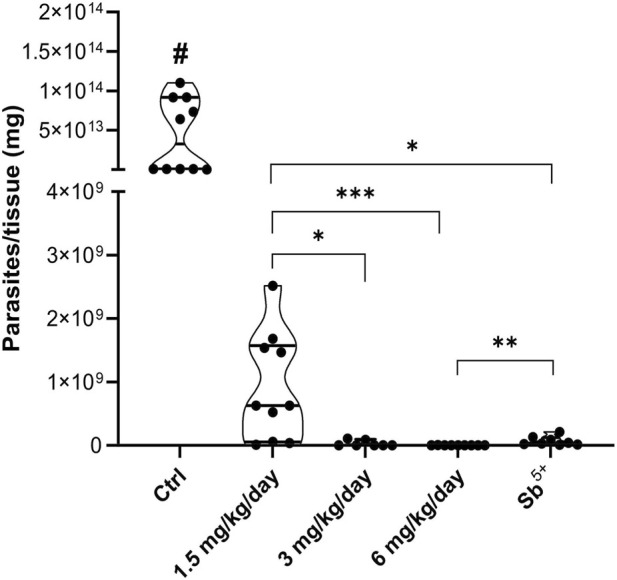
Long-term therapeutic effectiveness of memantine in a murine model of visceral leishmaniasis. BALB/c mice were infected with *Leishmania infantum* promastigotes via intraperitoneal injection. At 7 days post-infection, the mice were treated orally with 1.5, 3, or 6 mg/kg/day of memantine (treated group) or vehicle (control group) or intramuscularly injected with 100 mg/kg/day of meglumine antimoniate (positive control group). The mice were euthanized 18 days after the end of treatment, and the livers were collected to quantify parasite load by using a limiting dilution assay (LDA). A significant difference between control and treated groups is indicated by #p ≤ 0.0001; significant differences between the treated groups are indicated by *p ≤ 0.05, **p ≤ 0.005, and ***p ≤ 0.0005. The values are presented as the means ± standard errors of 2 independent experiments with 5 animals per group. Student’s t test with the Mann‒Whitney *post hoc* test was used for the analysis. Ctrl, control; Sb^5+^, meglumine antimoniate.

Under the long-term scheme (Figure 4), all memantine doses reduced the liver parasite load by 99% compared with the vehicle control. Similarly, in the long-term scheme, a clear trend of dose-related efficacy was observed, with the lowest dose (1.5 mg/kg/day) being less effective than the intermediate and highest doses. Compared with the meglumine antimoniate group, the 6 mg/kg/day memantine group showed superior parasite clearance and a significantly lower hepatic parasite burden.

Spleen parasite loads were also evaluated. In the short-term scheme, all memantine-treated groups showed significantly lower parasite burdens than the meglumine antimoniate group, and memantine showed superior effectiveness even at the lowest dose. The reduction in the parasite load exceeded 90% for the 3 and 6 mg/kg/day doses, while the 1.5 mg/kg/day dose achieved a 78.2% reduction in the parasite load, which was similar to that of meglumine antimoniate (79.1%) (Supplementary Figure S2).

Under the long-term scheme, memantine treatment resulted in a 99% reduction in the spleen parasite burden at all doses. The 6 mg/kg/day dose was significantly more effective than all other treatments, including meglumine antimoniate. This difference was more pronounced in the long-term scheme, highlighting the increased therapeutic potential of higher doses during prolonged treatment (Supplementary Figure S3).

### 3.3 The IFN-γ/IL-10 balance in infected mice is modulated by memantine treatment

**FIGURE 5:**
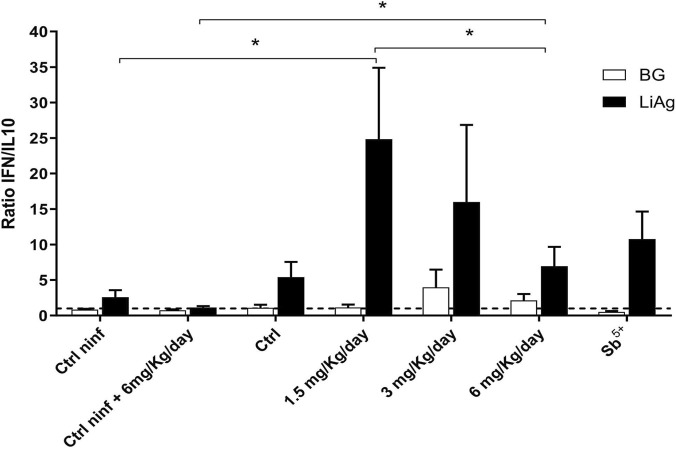
Modulation of the IFN-γ/IL-10 balance by memantine treatment in *Leishmania infantum*-infected mice. Splenocytes from infected and uninfected mice were stimulated *in vitro* with LiAg for 72 h, and cytokine concentrations were measured by using a cytometric bead array (CBA). Data are presented as the IFN-γ/IL-10 ratio, with values above 1 (dotted line) indicating a Th1-skewed profile. The data are presented as means ± standard deviations (SDs) obtained in two independent experiments. *p ≤ 0.05. Ctrl, control; Ctrl ninf, noninfected control; Sb^5+^, meglumine antimoniate.

An analysis of IFN-γ/IL-10 ratios in infected mice revealed dose-dependent immunological modulation. Ratios >1 indicated the predominance of IFN-γ, which is consistent with a Th1-skewed immune response (Figure 5). The untreated infected controls had ratios near or <1, reflecting similar levels or a predominance of IL-10, which was associated with immunosuppression and leishmaniasis chronicity. Memantine-treated groups consistently exhibited increased IFN-γ production compared with that of the control group. The meglumine antimoniate group showed a similar, but less prominent, pattern. While not statistically significant, a trend was observed for the 1.5 mg memantine group, with greater IFN-γ/IL-10 dispersion, and the 6 mg group, with less dispersion but lower ratios, possibly indicating a more regulated response (Figure 5).

Uninfected animals treated with 6 mg/kg/day memantine were compared to uninfected, untreated controls to investigate the infection-independent immunomodulatory effects. No significant modulation was observed, with both groups showing an IFN-γ/IL-10 ratio near 1. In contrast, compared with the uninfected treated group, the infected group treated with 6 mg/kg/day memantine exhibited a statistically significant increase in the IFN-γ/IL-10 ratio (Figure 5).

### 3.4 Evaluation of nitric oxide production in spleen cells

**FIGURE 6:**
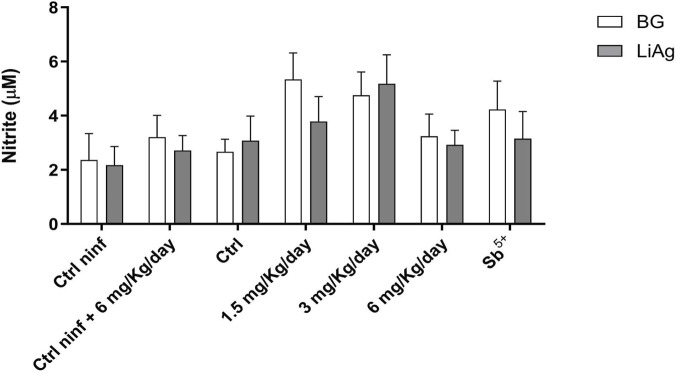
Nitrite production by spleen cells stimulated with or without *Leishmania* antigen (LiAg). Spleen cells were obtained from the supernatant of spleen macerates from mice that were either noninfected or infected with *Leishmania infantum* and treated with or without memantine or meglumine antimoniate under a long-term treatment regimen. Cells were either unstimulated (BG) or stimulated with soluble *Leishmania* antigen (LiAg) for 72 h. After the incubation, nitric oxide (NO) production was assessed indirectly by measuring nitrite levels in the culture supernatants using the Griess assay. The data are presented as the means ± standard deviations (SDs) from two independent experiments, each using five animals per group. Ctrl, control; Ctrl ninf, noninfected control; BG, background group (unstimulated); LiAg, *Leishmania infantum* antigen.

Nitric oxide (NO) levels in the splenic cell culture supernatants were quantified. Compared with untreated infected controls, infected animals treated with 1.5 mg/kg/day and 3 mg/kg/day showed a discrete, nonstatistically significant increase in NO production, suggesting a trend toward macrophage activation. Animals treated with 6 mg/kg/day memantine exhibited reduced NO levels, similar to those in the meglumine antimoniate group (Figure 6).

### 3.5 The correlation of the parasite load with cytokine production indicates a possible *immunomodulatory role of memantine*

**FIGURE 7:**
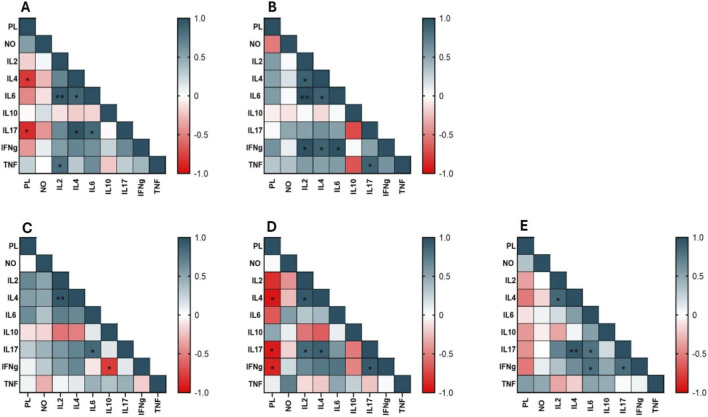
*Heatmap* of the Spearman correlation coefficients between the parasite load and the levels of nitric oxide and pro- and anti-inflammatory cytokines. **(A)** Control; **(B)** meglumine antimoniate; **(C)** 1.5 mg/kg/day memantine; **(D)** 3 mg/kg/day memantine; **(E)** 6 mg/kg/day memantine. The analysis was performed by determining the correlations between cytokine levels and *Leishmania infantum* infection (parasite load). Warm colors indicate negative correlations, while cool colors indicate positive correlations. Spearman’s correlation coefficients (p) are represented by the color scale, with statistical significance indicated by asterisks (*p ≤ 0.05 and **p ≤ 0.005). PL, parasite load; NO, nitric oxide.

Spearman’s correlation analysis revealed dynamic and group-specific cytokine interaction patterns. In the infected, untreated control animals (Figure 7A), the negative correlation between the parasite load and the levels of IL-4 and IL-17 suggested that increased expression of these cytokines might represent unsuccessful or late compensatory immune activation. IL-2 expression was positively correlated with IL-6 and TNF-α expression, as well as with IL-4 and IL-17 expression. IL-6 and IL-17 levels were also positively correlated, indicating that a coordinated but likely ineffective proinflammatory environment involving the Th2 and Th17 axes was insufficient for parasite clearance.

In the meglumine antimoniate-treated group (Figure 7B), the correlation network reflected a stronger functional and protective response. IL-2 levels correlated positively with IL-4, IL-6, and IFN-γ levels, while IL-4 and IL-6 levels also correlated with IFN-γ levels. IL-17 levels correlated positively with TNF-α levels. These associations reflect coordinated Th1 and Th17 activation, supported by regulatory modulation, which is consistent with a low parasite burden.

After treatment with 1.5 mg/kg memantine (Figure 7C), the correlation matrix revealed a mixed but relatively balanced response. A significant negative correlation between IL-10 and IFN-γ levels suggested that reduced IL-10 levels might increase the effectiveness of Th1 activity. Positive correlations between IL-2 and IL-4 levels and between IL-6 and IL-17 levels indicated the coexistence of Th2 and Th17 components. This profile suggests a functional but intermediate response, with partial parasite control and a median parasite burden.

Treatment with the 3 mg/kg dose (Figure 7D) produced the most diverse and integrated correlation network. The parasite load was negatively correlated with the levels of IL-17, IL-4, and IFN-γ, confirming their association with improved parasite control. Strong positive correlations were observed between IL-2 and IL-4 levels, between IL-2 and IL-17 levels, between IL-4 and IL-17 levels, and between IFN-γ and IL-17 levels. These findings indicate a highly effective immune response driven by synergistic Th1 and Th17 activation, with IL-4 present but not deleterious. The parasite burden ranged from low to intermediate, reflecting variability but overall improved control.

In the 6 mg/kg group (Figure 7E), the correlations were weaker, especially for IL-17 and IL-4 levels, but this group exhibited the lowest parasite burden (Figure 4). Positive associations were observed between IL-2 and IL-4, IL-4 and IL-17, IL-6 and IL-17, IL-6 and IFN-γ, and IL-17 and IFN-γ levels, suggesting broad but regulated immune activation. The overall pattern reflects a multifunctional response integrating Th1, Th2, and Th17 pathways in a balanced manner, supporting parasite elimination with minimal reliance on intense inflammatory signaling.

**FIGURE 8:**
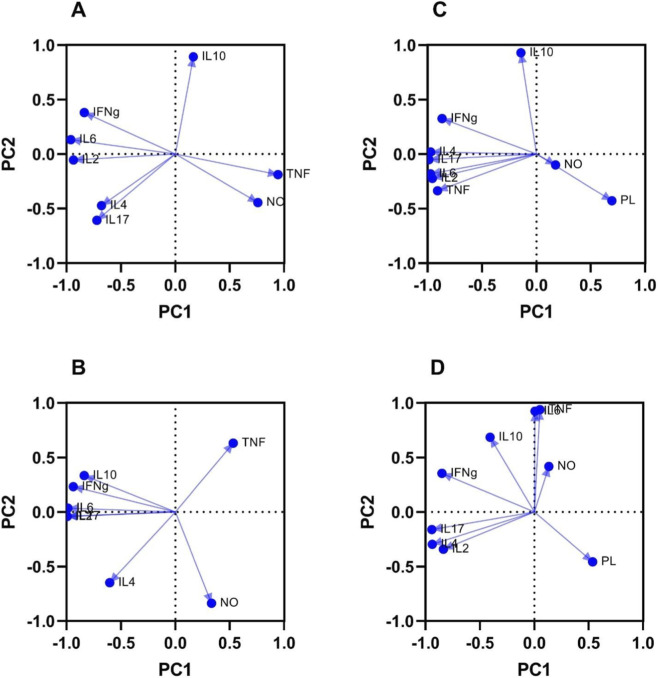
Principal component analysis (PCA) of the cytokine profiles in the supernatants of splenocyte cultures stimulated *in vitro* with LiAg. **(A)** Noninfected mice. **(B)** Noninfected mice were treated with 6 mg/kg/day memantine. **(C)** Infected untreated mice. **(D)** Infected mice were treated with 6 mg/kg/day memantine. The direction and length of a vector indicate how a cytokine or immune mediator contributes to the two principal components in the plot. PC1 separates regulatory (IL-10-driven) from effector Th1/Th17-associated responses, whereas PC2 reflects differences in the coordination and intensity of cytokine activation. PL, parasite load; NO, nitric oxide.

The results of principal component analysis (PCA) of cytokine responses from splenocytes stimulated *in vitro* with LiAg highlighted the immunomodulatory action of memantine under both noninfected and infected conditions. In noninfected animals (Figure 8A), cytokines exhibited a basal, nonpolarized pattern dominated by IL-10. Treatment with 6 mg of memantine (Figure 8B) shifted this profile, with TNF and nitric oxide (NO) strongly contributing to the main component and IL-10 losing prominence, which is consistent with enhanced Th1/Th17-like responsiveness following memantine treatment alone.

In infected untreated mice (Figure 8C), IL-10 remained the major source of variation and was segregated from IFN-γ, TNF, and NO, consistent with an uncoordinated, immunosuppressive response that is typical of active VL. In contrast, infected animals treated with memantine (Figure 8D) displayed a reorganized cytokine network, with IFN-γ, IL-2, IL-17, and TNF clustering together, while the level of IL-10 shifted away, and the parasite load was inversely related to the levels of these effector cytokines. Overall, PCA clearly demonstrated that memantine restored coordination among protective immune mediators and promoted antigen-specific activation even in uninfected hosts.

## 4 Discussion

VL remains a significant global health problem, causing thousands of deaths annually, particularly in vulnerable populations. When left untreated, its severe clinical form leads to over 90% mortality (Rijal et al., 2019; Costa et al., 2023). Current VL chemotherapy faces substantial drawbacks, including high toxicity, complex administration, cost, and increasing drug resistance (Rijal et al., 2019; van Griensven and Diro, 2019; Mathison and Bradley, 2023), necessitating new, effective, safe, and accessible therapeutic strategies.

Drug repurposing represents a practical alternative to conventional drug development, which is lengthy and costly (Pushpakom et al., 2019). By leveraging compounds with established safety and pharmacokinetic profiles, repurposing reduces risks and accelerates clinical translation (Andrade-Neto et al., 2018; Ferreira et al., 2022). Historically, many VL drugs have been repurposed (Burza et al., 2018), validating this strategy for neglected tropical diseases.

Memantine, an uncompetitive NMDA receptor antagonist, prevents glutamate excitotoxicity (Danysz et al., 2000). While primarily known for their role in the CNS (Paoletti et al., 2013), NMDA receptors are also found in peripheral tissues and immune cells (Gonda, 2012). Memantine shows effectiveness against *Trypanosoma cruzi* but has not been evaluated against *Leishmania* (Damasceno et al., 2014; Souza et al., 2019).

Our study demonstrated the significant antileishmanial activity of memantine, with an IC_50_ of 5.49 ± 0.11 μM against *L. infantum* intracellular amastigotes and a high selectivity index of 603.64. In *L. infantum*-infected BALB/c mice, oral memantine treatment (1.5, 3, and 6 mg/kg/day) markedly reduced hepatic and splenic parasite loads (>99%) after both short-term and long-term regimens. The administration of 6 mg/kg/day dose outperformed the treatment with meglumine antimoniate. Importantly, memantine treatment did not induce adverse effects. This dual effectiveness, achieving high parasite clearance across various treatment periods, compares favorably with existing therapies (Rijal et al., 2019).

Memantine fulfills the established criteria for new leishmaniasis drug candidates: an IC_50_ < 10 μM, an SI ≥ 10, and a >70% reduction in the liver parasite load *in vivo* (Pink et al., 2005; Katsuno et al., 2015). Its superior effectiveness and high selectivity index compare favorably to those of meglumine antimoniate and miltefosine, which have toxicity and resistance issues (Dorlo et al., 2012; Reimão et al., 2020). Oral administration and the lack of toxicity of memantine at the tested doses suggest that it provides significant logistical and therapeutic advantages over parenteral treatments.

While nitric oxide (NO) is crucial for parasite destruction (Ferreira-Paes et al., 2020; Formaglio et al., 2021), NO levels in splenocyte cultures did not markedly differ. These findings suggest that higher doses of memantine achieve parasite clearance via alternative mechanisms, such as cytokine modulation and restoration of the immune balance, rather than increased nitrosative stress, which conforms with the reduced role of NO in chronic VL (Bhor et al., 2021; Na et al., 2024).

VL progression is mainly influenced by the host immune response, with disease control depending on the balance between effector and regulatory mechanisms (Gautam et al., 2014). VL susceptibility is linked to a Th2-type response (IL-4, IL-10, and IL-13), whereas resistance is associated with Th1 cytokines (IFN-γ and IL-12) (Rossi and Fasel, 2018; Goto and Mizobuchi, 2023). Effective *L. infantum* control requires a coordinated Th1 response, with IFN-γ and TNF-α activating macrophages for NO-mediated killing (Bhor et al., 2021; Na et al., 2024; Tiwari et al., 2024). Chronic VL, however, is characterized by immune dysfunction, including reduced IFN-γ levels and elevated IL-10/TGF-β levels, promoting parasite persistence.

Our study revealed that memantine induces a dose-dependent immunomodulatory effect that influences cytokine patterns and parasite control. Memantine-treated groups presented higher IFN-γ/IL-10 ratios, supporting a favorable immunological balance by enhancing Th1 effector responses while restraining IL-10-mediated suppression. The lowest dose (1.5 mg/kg/day) resulted in an intermediate immune profile, with negative correlations between IL-10 and IFN-γ levels, suggesting periods of effective Th1-mediated parasite control, whereas positive IL-2/IL-4 associations suggested partial Th2 activation. These changes reflect a transitional state toward resistance, highlighting that even low-dose memantine can initiate protective immune modulation. The 3 mg/kg/day dose produced the most robust immunoprotective profile, with synergistic activation of the Th1, Th2, and Th17 pathways being observed, which is consistent with effective parasite control through a coordinated and functional inflammatory network (Na et al., 2024; Tiwari et al., 2024).

The highest dose (6 mg/kg/day) effectively controlled parasitemia with a stable, interconnected cytokine network, suggesting a multifaceted but controlled immune activation that maintains parasite suppression with minimal inflammatory stress, which is crucial in VL, where immune hyperactivation can exacerbate tissue damage (Bhor et al., 2021; Tiwari et al., 2024). The reduced NO levels observed after treatment are consistent with the involvement of NO-independent parasiticidal mechanisms. Furthermore, PCA demonstrated an immunomodulatory effect of memantine through the reorganization of cytokine networks in both infected and noninfected mice. Treatment with 6 mg/kg/day memantine was associated with a shift in the response from an IL-10-dominated profile to a coordinated Th1/Th17 pattern, thereby increasing IFN-γ, TNF-α, and IL-17 activity and reducing the immunosuppression that is a typical feature of VL. Previous studies support the immunomodulatory role of memantine, which upregulates IFN-γ and TNF-α while downregulating IL-10 (Willard et al., 2000; Wu et al., 2009; Rani et al., 2022).

Cytokine balance is central to VL outcomes. Th1 cytokines drive macrophage activation and parasite clearance, whereas immunosuppressive cytokines inhibit Th1 responses (Na et al., 2024). IL-6, in particular, promotes Th2 polarization and suppresses classical macrophage activation (Murray, 2008). High IL-6 levels in VL patients are associated with mortality (Costa et al., 2023). Conversely, IL-17, especially in conjunction with IFN-γ, increases NO production, induces neutrophil recruitment, and supports granuloma formation (Kumar et al., 2019).

Lymphocyte subsets also modulate VL. CD4^+^ Th1 cells are protective, whereas Th2 and Tr1 cells favor persistence. CD8^+^ T cells can be protective but often become exhausted (Esch et al., 2013; Gautam et al., 2014, Habib et al., 2018). Our findings suggest that memantine helps restore a protective immune environment, potentially by modulating these cellular responses, although further investigation is needed to fully elucidate these mechanisms.

## 5 Conclusion

Overall, the results of this study successfully demonstrate the significant antileishmanial activity of memantine both *in vitro* against *L. infantum* intracellular amastigotes and *in vivo* in a murine model of VL. Memantine effectively reduced parasite loads in the liver and spleen, even outperforming meglumine antimoniate at higher doses, without inducing observable toxicity. Furthermore, our findings highlight the immunomodulatory properties of memantine, as it can restore a protective immune environment characterized by an enhanced Th1 response and controlled cytokine balance. These results greatly support memantine as a promising candidate for drug repurposing in the treatment of VL, offering a potentially safer, orally administered, and effective alternative to current therapies. Further research is warranted to elucidate its mechanisms of action and advance its clinical development as a treatment for this neglected tropical disease.

## Data Availability

The raw data supporting the conclusions of this article will be made available by the authors, without undue reservation.
