# Supplementary material for: Repurposing memantine as an oral therapy for visceral leishmaniasis: identification of direct leishmanicidal activity and immune system modulation in preclinical studies
Source: Front Pharmacol. 2026 Mar 30;17:1761504. doi: 10.3389/fphar.2026.1761504 (PMC13070929; doi:10.3389/fphar.2026.1761504)
Supplement: Supplementary file 1 [file DataSheet2.pdf]

Supplemental material

Supplementary Figure 1. Sigmoidal concentration-response curve for memantine against *Leishmania infantum* amastigotes. The sigmoidal dose-response curve shows the inhibitory effect of memantine on intracellular *Leishmania infantum* amastigotes in macrophage cultures. *L. infantum*-infected macrophages were incubated for 72 hours with varying concentrations of memantine (0.64–2.44 µM) expressed in log (logarithmic scale). All values represent the means ± standard errors from three independent experiments (n=3) performed in duplicate. A significant difference compared to the control is indicated by * $p < 0.0001$. The curve was fitted using non-linear regression analysis. The IC_{50} value was estimated at 5.49 µM, corresponding to a log concentration of 1.74. Asterisks (*) indicate statistical significance compared to the control ($p < 0.05$).

Supplementary Figure 2. Parasite load in the spleen of the murine model of visceral leishmaniasis treated with memantine under the short-term scheme. BALB/c mice were infected with *L. infantum* promastigotes via intraperitoneal injection. Seven days after the infection, the groups were treated orally with 1.5, 3, or 6 mg/kg/day of memantine or vehicle or intramuscularly injected with 100 mg/kg/day of meglumine antimoniate. The mice were euthanized at the end of treatment, and the spleens were collected to quantify the parasite load using a limiting dilution assay (LDA). A significant difference between control and treated groups is indicated by # $p \leq 0.0001$; Significant differences between the treated groups are indicated by* $p \leq 0.05$ and ** $p \leq 0.005$. The values are presented as the means ± standard errors of 1 independent experiment with 5 animals per group. Student's *t* test with the Mann–Whitney post hoc test were used for the analysis. Ctrl, control; Sb^{5+}, meglumine antimoniate.

Supplementary Figure 3. Parasite load in the spleen of murine model of visceral leishmaniasis treated with memantine under the long-term scheme. BALB/c mice were infected with *L. infantum* promastigotes via intraperitoneal injection. Seven days after the infection, the groups were treated orally with 1.5, 3, or 6 mg/kg/day of memantine or vehicle or intramuscularly injected with 100 mg/kg/day of meglumine antimoniate. The mice were euthanized 18 days after the end of treatment, and the spleens were collected to quantify the parasite load using a limiting dilution assay (LDA). A significant difference between control and treated groups is indicated by # $p \leq 0.0001$; Significant differences between the treated groups are indicated by * $p \leq 0.05$

and ** $p \leq 0.005$. The values are presented as the means ± standard errors of 1 independent experiment with 5 animals per group. Student's *t* test with the Mann–Whitney post hoc test were used for the analysis. Ctrl, control; Sb^{5+}, meglumine antimoniate.

Supplementary Table 1. Biochemical parameters measured at the end of the experiment. After 5 days of treatment, the BALB/c mice were anesthetized, blood was collected, and serum was separated for the analysis of biochemical markers. AST = aspartate aminotransferase; ALT = alanine aminotransferase. The values are presented as the means ± standard errors of the experiment with 5 animals in each group.

Biochemical parameters were measured using the clinical analysis platform of Instituto de Ciência e Tecnologia em Biomodelos (FIOCRUZ, BR).

Supplementary Table 2. Hematological parameters measured at the end of the experiment. After 5 days of treatment, the BALB/c mice were anesthetized, and blood was collected. RBCs = red blood cells; MCV = mean corpuscular volume; MCH = mean corpuscular hemoglobin; MCHC = mean corpuscular hemoglobin concentration. The values are presented as the means ± standard errors of the experiment with 5 animals in each group. Hematological parameters were measured using the clinical analysis platform of Instituto de Ciência e Tecnologia em Biomodelos (FIOCRUZ, BR).
