# Supplementary figures and images for: Repurposing memantine as an oral therapy for visceral leishmaniasis: identification of direct leishmanicidal activity and immune system modulation in preclinical studies
Source: Front Pharmacol. 2026 Mar 30;17:1761504. doi: 10.3389/fphar.2026.1761504 (PMC13070929; doi:10.3389/fphar.2026.1761504)

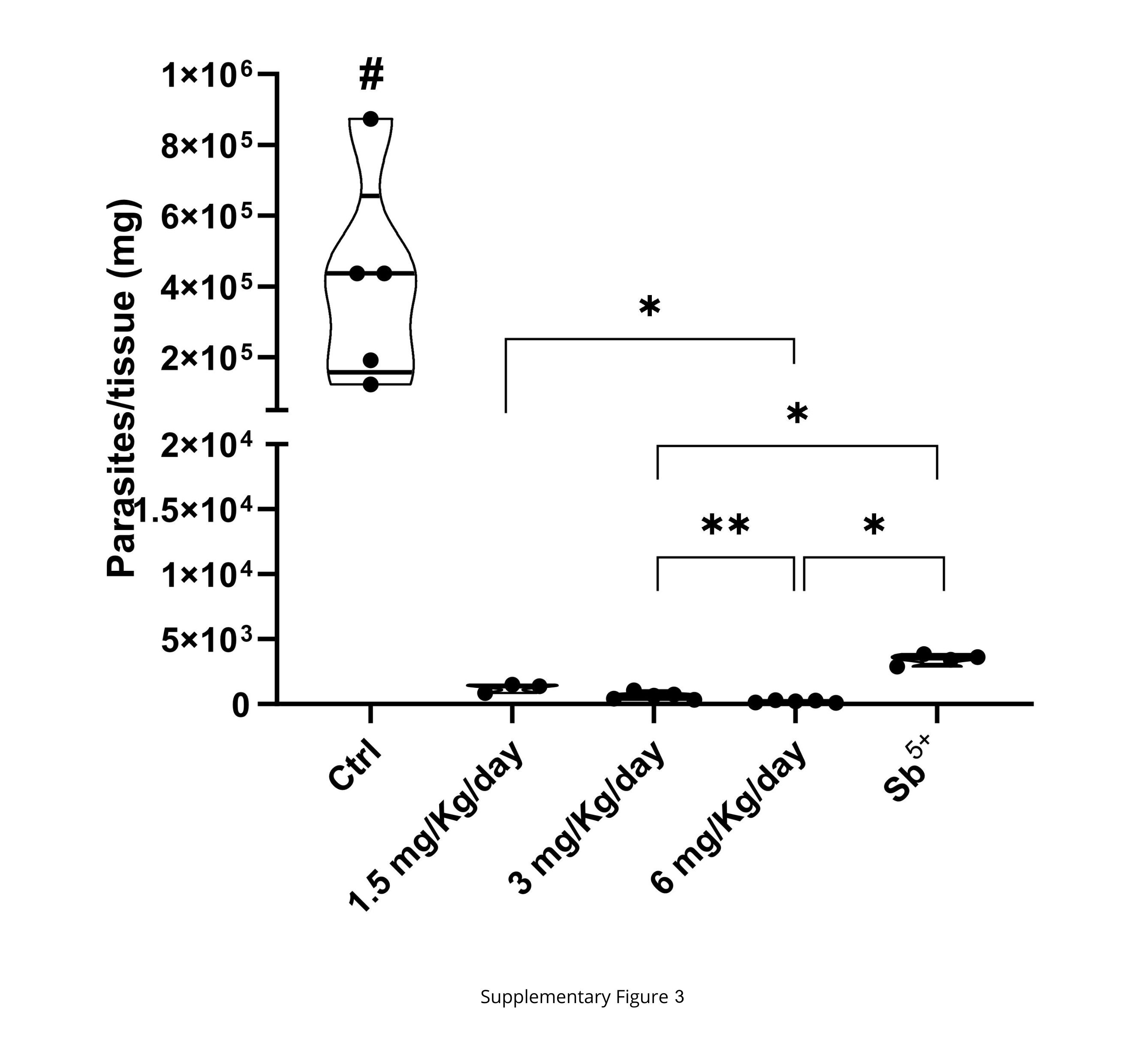

Supplement: Supplementary file 2 [file Image3.jpg]

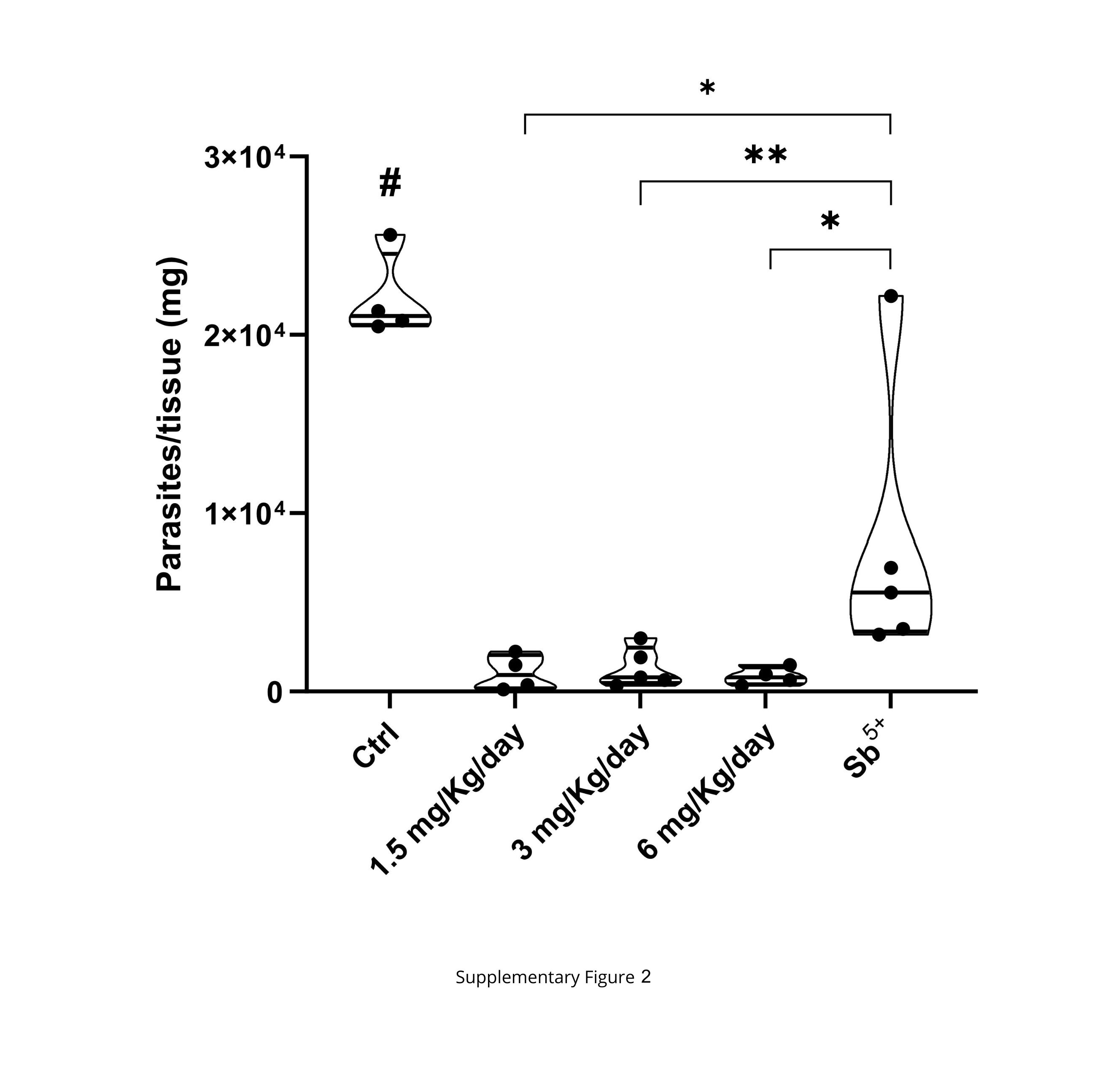

Supplement: Supplementary file 3 [file Image2.jpg]

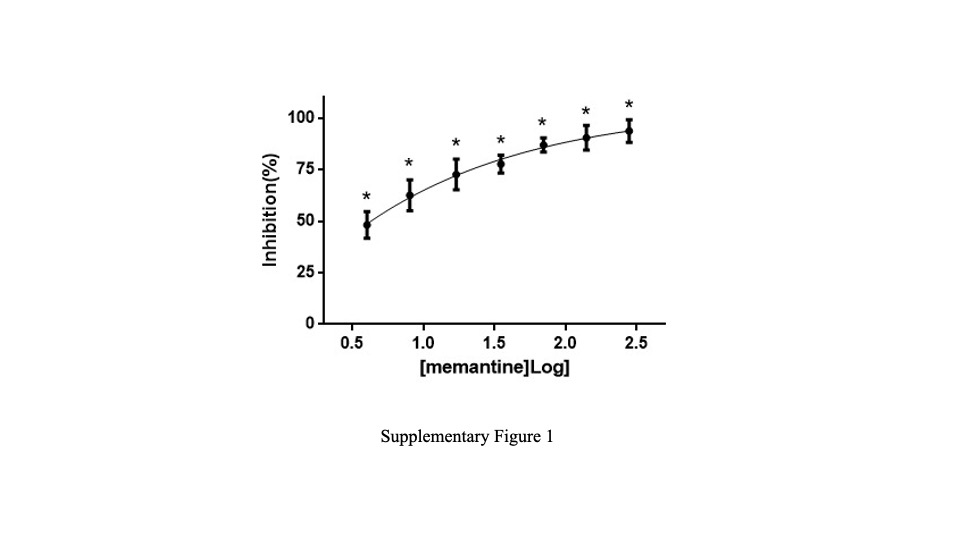

Supplement: Supplementary file 4 [file Image1.jpeg]
