# Supplementary material for: Repurposing memantine as an oral therapy for visceral leishmaniasis: identification of direct leishmanicidal activity and immune system modulation in preclinical studies
Source: Front Pharmacol. 2026 Mar 30;17:1761504. doi: 10.3389/fphar.2026.1761504 (PMC13070929; doi:10.3389/fphar.2026.1761504)
Supplement: Supplementary file 5 [file DataSheet1.pdf]

Supplementary Table 1. Biochemical parameters measured at the end of the experiment.

	Reference values	Control	1.5 mg/Kg/day	3 mg/Kg/day	6 mg/Kg/day	Meglumine antimoniate
**Sodium (mEq/L)**	157 – 174	166 ± 1.56	168 ± 0.82	162 ± 1.13	160 ± 0.25	165 ± 0.93
**Potassium (mEq/L)**	4.6 – 8	7.1 ± 0.73	7.3 ± 0.61	7.4 ± 1.23	6.3 ± 1.48	7.4 ± 1.52
**Urea (mg/dL)**	18 – 29	28.7 ± 0.08	27.7 ± 0.67	27.1 ± 0.32	27.5 ± 0.79	28.4 ± 1.09
**Albumin (mg/dL)**	2.5 – 4.8	2.9 ± 0.43	4.2 ± 0.03	4.1 ± 0.08	3.9 ± 0.51	3.6 ± 1.11
**Calcium (mg/dL)**	5.9 – 9.4	6.2 ± 0.12	6.9 ± 0.59	7.2 ± 1.25	7.3 ± 1.54	6.1 ± 1.57
**Iron (mg/dL)**	130 – 134	132.5 ± 1.27	132.4 ± 0.07	130.1 ± 0.02	130.9 ± 0.06	132.9 ± 0.34
**AST**	59 – 247	222 ± 0.04	193.1 ± 1.62	152.5 ± 2.26	196 ± 0.39	186 ± 1.27
**ALT**	28 – 132	86.3 ± 2.36	92.1 ± 3.09	72.3 ± 2.89	84.3 ± 1.11	88.7 ± 1.09
**Creatine Kinase**	68 – 1070	285 ± 3.4	589 ± 8.23	326 ± 1.27	514 ± 10.2	566 ± 9.3
**Alkaline Phosphatase**	62 – 209	57.2 ± 0.31	61.1 ± 0.97	68.2 ± 0.66	61.1 ± 1.89	63.1 ± 1.56
**Cholesterol (mg/dL)**	36 – 96	101 ± 7.14	92.3 ± 1.02	92 ± 0.04	96.7 ± 2.54	99.1 ± 0.51
**Total proteins (g/dL)**	3.6 – 6.6	4.2 ± 0.16	4.3 ± 0.47	4.6 ± 1.23	4.9 ± 0.31	4.9 ± 0.09

After 5 days of treatment, the BALB/c mice were anesthetized, blood was collected, and serum was separated for the analysis of biochemical markers. AST = aspartate aminotransferase; ALT = alanine aminotransferase. The values are presented as the means ± standard errors of the experiment with 5 animals in each group. Biochemical parameters were measured using the clinical analysis platform of Instituto de Ciência e Tecnologia em Biomodelos (FIOCRUZ, BR).

Supplementary Table 2. Hematological parameters measured at the end of the experiment.

	Reference values	Control	1.5 mg/Kg/day	3 mg/Kg/day	6 mg/Kg/day	Meglumine antimoniate
**RBC (mil/mm^3)**	8.2 – 10.2	8.7 ± 0.13	9.6 ± 0.09	8.9 ± 0.07	9.5 ± 0.02	9.8 ± 0.12
**Hemoglobin (g/dL)**	14.4 – 15.7	14.9 ± 0.03	14.7 ± 0.15	15.1 ± 0.15	14.2 ± 0.32	14.2 ± 0.32
**Hematocrit (%)**	46.18 – 58.42	58.3 ± 0.23	53.9 ± 0.06	52.3 ± 0.22	53.6 ± 0.10	56.3 ± 0.26
**MCV (fL)**	41.3 – 55.5	50.7 ± 0.44	50.9 ± 0.64	46.6 ± 0.08	51.9 ± 1.28	44.9 ± 1.46
**MCH (pg)**	14.9 – 16.5	15.4 ± 0.15	15.2 ± 0.10	14.9 ± 0.53	15.4 ± 0.58	15.3 ± 0.16
**MCHC (g/dL)**	27.4 - 32	31.2 ± 1.16	28.3 ± 0.97	30.6 ± 0.65	28.9 ± 0.65	28.9 ± 0.08

After 5 days of treatment, the BALB/c mice were anesthetized, and blood was collected. RBCs = red blood cells; MCV = mean corpuscular volume; MCH = mean corpuscular hemoglobin; MCHC = mean corpuscular hemoglobin concentration. The values are presented as the means ± standard errors of the experiment with 5 animals in each group. Hematological parameters were measured using the clinical analysis platform of Instituto de Ciência e Tecnologia em Biomodelos (FIOCRUZ, BR).
